# Design of Superhydrophobic CoFe_2_O_4_ Solar Seawater Desalination Device and Its Application in Organic Solvent Removal

**DOI:** 10.3390/nano12091531

**Published:** 2022-05-02

**Authors:** Xiangcai Ge, Zhijun Zhou, Zheng Tan, Shoufei Wang, Xingchuan Zhao, Guina Ren, Bo Ge, Wei Li

**Affiliations:** 1 School of Materials Science and Engineering, Liaocheng University, Liaocheng 252059, China; gexiangcai@lcu.edu.cn (X.G.); 2020205938@stu.lcu.edu.cn (Z.Z.); 2019205643@stu.lcu.edu.cn (Z.T.); 2018206124@stu.lcu.edu.cn (S.W.); liwei@lcu.edu.cn (W.L.); 2 School of Environmental and Material Engineering, Yantai University, Yantai 264405, China; guina.ren@ytu.edu.cn

**Keywords:** superhydrophobic, oil–water separation, clean water production

## Abstract

Environmental pollution and clean water production are challenges to the development of human society. In this paper, devices consisting of a superhydrophobic Ni-CoFe_2_O_4_ foam layer (floating layer), a hydrophilic channel and a superhydrophilic Ni-CoFe_2_O_4_ foam layer (photothermal conversion layer) were designed. The light energy was converted into heat on the photothermal layer, for which the hydrophilic channel provided a small amount of water. The superhydrophobic layer ensured the floating and selective adsorption of organic solvents on the water surface, whose contact angle reached 157°, and the steam production rate reached 1.68 kg·m^−2^·h^−1^. Finally, the LSV curve demonstrated that the Ni-CoFe_2_O_4_ foam prepared had a minimum starting potential, achieving the multifunctionality of the Ni foam.

## 1. Introduction

Environmental and drinking water safety is a global challenge that hinders social development [1,2]. Statistics show that water consumption will grow by >55% by 2050 with the increase in GDP [3]. Desalination is an important way to solve water shortage. At present, desalination methods mainly include thermal and membrane separation methods (RO), accounting for more than 90% of the market. Traditional RO methods have an energy consumption of 3.5 kWh_elec_/m^3^ [4]. The enthalpy of evaporation (∆H) needs to be overcome for thermal methods, and the separation energy (∆G) needs to be overcome for filtration methods. ∆H is two orders of magnitude higher than ∆G [5]. Compared with seawater desalination methods that consume fossil fuels, only solar energy is used in solar desalination to produce clean water, which has attracted wide attention from researchers [6,7]. Through the modification of polydopamine and Ti_3_C_2_T_x_ Mxenes, Chen et al. [8] used balsa wood as a raw material to obtain an evaporator, which had a high steam production speed of 2.08 kg·m^−2^·h^−1^. Lin et al. [9] loaded poly(N-phenylglycine) and MoS_2_ on a polyvinylidene fluoride (PVDF) film to form a photothermal layer. A steam production rate of 1.7 kg·m^−2^·h^−1^ was obtained for the photothermal device, assisted by hydrophilic channels and polystyrene foam. When photothermal technology is applied to sewage discharged from chemical plants, the problem of floating organic solvent pollutants needs to be solved. Superhydrophobic–superoleophilic materials have attracted research interests due to their ability to selectively adsorb organic solvents [10,11]. Liu et al. [12] prepared superhydrophobic silica floating on the oil–water interface, which could selectively adsorb the oil phase to achieve the purpose of oil–water separation. However, superhydrophobic silica is difficult to recover from the powder state.

In order to realize floating oil removal and clean water production, a multifunctional solar evaporator was designed, which is composed of a superhydrophobic floating layer, a filter paper layer and a CoFe_2_O_4_ photothermal layer. The superhydrophobic layer has two functions: one is to selectively adsorb floating oil and the other is to support the floating of the device. Through the synergy of the three layers, a solar evaporation rate of 1.68 kg·m^−2^·h^−1^ was obtained by the device, providing a new solution for the application of solar evaporators in sewage treatment.

## 2. Experimental Section

### 2.1. Material

HCl (AR, 36–38 wt %), acetone (AR, 99.5%) and ethanol (AR, 99.7%) were supplied by Yantai Far East Fine Chemical Co., Ltd. FeCl_2_ 4H_2_O (AR, 99.7%) was purchased from Tianjin Fuchen Chemical Reagents Factory. Co(NO_3_)_2_ 6H_2_O (AR, 99%), NHF (AR, 98%), CO(NH_2_)_2_ and Trichloro(1H, 1H, 2H, 2H-tridecafluoro-n-octyl)silane (AR, 97%) were provided by Aladdin Chemical Reagent Co., Ltd. Nickel foam was purchased from a local store.

### 2.2. Preparation of Solar Evaporator

The nickel foam was cut to the size of 3.5 cm × 3.5 cm and sonicated in 3 mol/L HCl, deionized water, acetone and ethanol for 20 min each. Amounts of 0.1592 g of FeCl_2_•4H_2_O, 0.4656 g of Co(NO_3_)_2_•6H_2_O, 0.0592 g of NH_4_F and 0.2402g of urea were dissolved in 65 mL of deionized water and transferred to a 100 mL stainless-steel autoclave. Nickel foam was added, reacting at 140 °C for 8 h. After cooling, the samples were dried at 140 °C for 0.5 h and then sintered at 400 °C for 3 h (heating speed was 2 °C/min). The resulting superhydrophilic Ni-CoFe_2_O_4_ foam was named the photothermal conversion layer. The floating layer was prepared as follows: the superhydrophilic Ni/CoFe_2_O_4_ foam was added to 30 mL of silane ethanol solution (0.5 mL) and stirred at room temperature for 4 h. A superhydrophobic Ni-CoFe_2_O_4_ foam floating layer was obtained after drying at 140 °C for 1 h. The structure of the solar evaporator was the superhydrophobic floating layer, the filter paper layer and the photothermal conversion layer from bottom to top.

### 2.3. Characterization

The structure of Ni, CoFe_2_O_4_ and superhydrophobic CoFe_2_O_4_ was obtained by TEM (FEI Tecnai F30) and XRD (Bruker D8w). The morphology of Ni, Ni-CoFe_2_O_4_ and superhydrophobic Ni-CoFe_2_O_4_ foams was obtained by FESEM (Zeiss Company, Ltd., Munich, Germany). The surface components of the superhydrophobic Ni-CoFe_2_O_4_ foam were obtained by XPS (ESC PHI500). The surface wettability of the foam was measured by a contact angle gauge (JC2000C1). The LSV curves of the foams were obtained via an electrochemical workstation (Gamry Reference 3000). The surface temperature of the Ni-CoFe_2_O_4_ foam was measured by an infrared camera (Fluke TiS20 + MAX).

## 3. Results and Discussion

**Figure 1:**
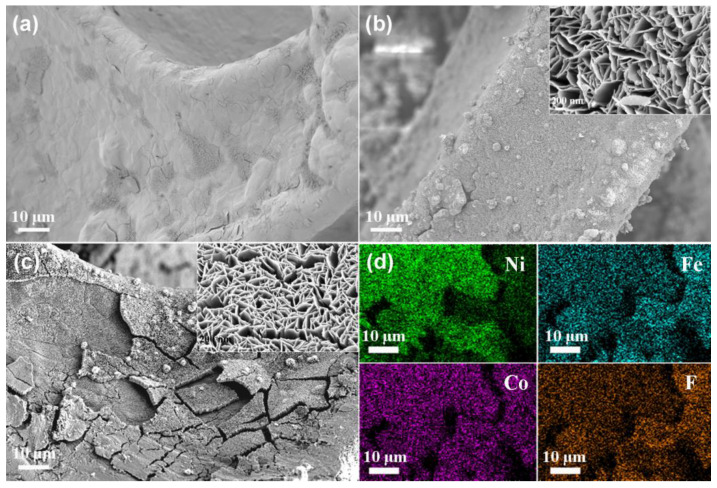
FESEM images of Ni foam (**a**), Ni-CoFe_2_O_4_ foam (**b**) and superhydrophobic Ni-CoFe_2_O_4_ foam (**c**). Element distribution of the superhydrophobic Ni-CoFe_2_O_4_ foam (**d**).

The flexibility and three-dimensional structure of nickel foam make it an excellent carrier. The rough surface of Ni foam was observed from its morphology (Figure 1a). The surface of the Ni foam where CoFe_2_O_4_ was grown became rougher. Importantly, on the basis of retaining the micron roughness, there was also nano roughness caused by the formation of nanosheets. Finally, micron-nano roughness was formed, which was beneficial to improving the hydrophobic performance (Figure 1b). No obvious change could be seen in the morphology of Ni-CoFe_2_O_4_ after the silane treatment (Figure 1c). The element distribution was investigated based on EDS analysis. Elements including Fe, Co and F had the same shape as the Ni foam, proving that superhydrophobic Ni-CoFe_2_O_4_ was successfully prepared (Figure 1d).

**Figure 2:**
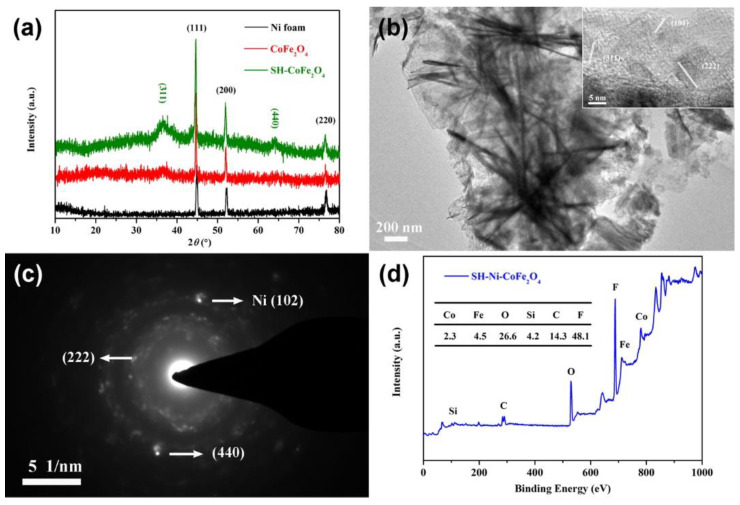
XRD analysis of CoFe_2_O_4_ composite materials (**a**). TEM morphology (**b**) and structure (**c**) analysis of CoFe_2_O_4_. XPS analysis (**d**) of superhydrophobic Ni-CoFe_2_O_4_.

To analyze the surface structure and composition of the composite, the results of XRD, TEM and XPS were analyzed. Notably, the CoFe_2_O_4_ powder used in the XRD and TEM analysis was obtained through ultrasonic dissection. As shown in Figure 2a, the characteristic peaks (111), (200) and (220) of Ni found in the diffraction peaks of both CoFe_2_O_4_ and superhydrophobic CoFe_2_O_4_ indicated that Ni particles were also stripped. The characteristic peaks (200) and (220) of CoFe_2_O_4_ appeared, proving that CoFe_2_O_4_ was formed on the surface of the Ni foam, and the crystal structure of CoFe_2_O_4_ was not changed through silane grafting. The nanosheet morphology of CoFe_2_O_4_ was confirmed in the TEM image, and the lattice planes (101), (311) and (222) were analyzed based on the HRTEM image (Figure 2b). The lattice plane (111) of Ni and the lattice plane (222) as well as (440) of CoFe_2_O_4_ were analyzed based on the SAED map (Figure 2c). As shown in Figure 2d, the surface of superhydrophobic CoFe_2_O_4_ is composed of elements Co, Fe, O, Si, C and F, and the content of F is up to 48.1%, which effectively reduces the surface energy.

**Figure 3:**
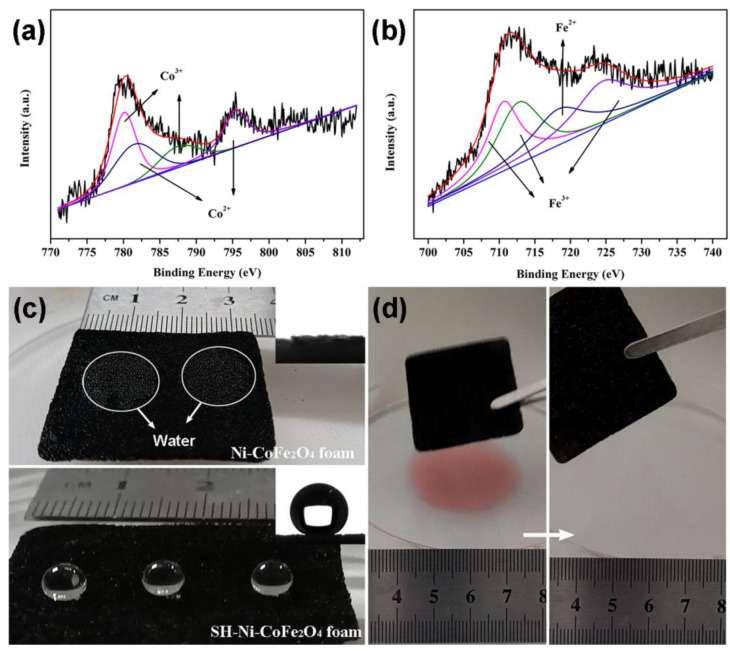
Fine spectrum of superhydrophobic Ni-CoFe_2_O_4_ (**a**,**b**). Image of the Ni-CoFe_2_O_4_ foam before and after silane modification (**c**). Selective organic solvent removal test for the Ni-CoFe_2_O_4_ foam (**d**).

Further fine spectral analyses of Co and Fe revealed that CoFe_2_O_4_ had a mixed valent state consisting of Co^2+^, Co^3+^, Fe^2+^ and Fe^3+^ (Figure 3a,b). Relevant information of the deconvolution is presented in Table S1. The superhydrophobic Ni-CoFe_2_O_4_ foam exhibited its superhydrophobicity under the synergy of the micron-nanoscale roughness and low surface energy. As shown in Figure 3c, the water droplets spread rapidly on the surface of the Ni-CoFe_2_O_4_ foam, showing their superhydrophilicity. The inset shows that the surface exhibited a contact angle of 0° on the water. After silane treatment, the surface of the superhydrophobic Ni-CoFe_2_O_4_ foam showed a non-wetting state with a contact angle of 157°. To verify the organic solvent removal property of the superhydrophobic Ni-CoFe_2_O_4_ foam, cyclohexane was used as the target organic solvent. When the superhydrophobic Ni-CoFe_2_O_4_ foam contacted the interface between cyclohexane and the water, cyclohexane was adsorbed, leaving a blank water surface.

**Figure 4:**
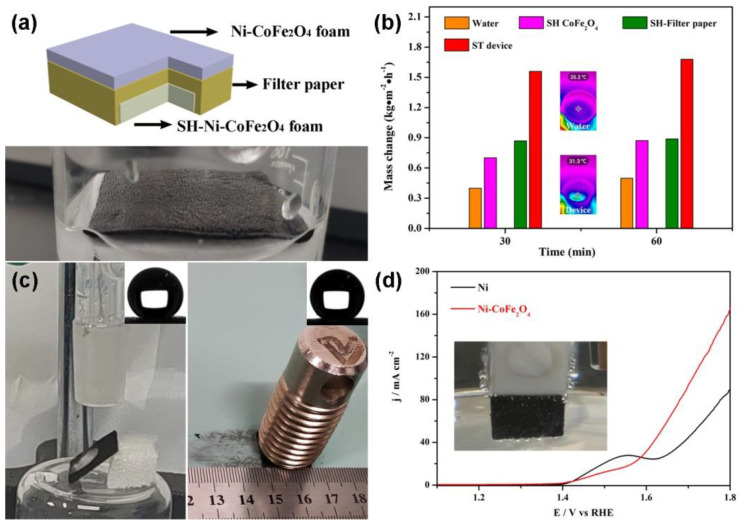
Schematic image of the solar steam device (**a**). Comparative experiment on the velocity of steam production (**b**). Stability test of the superhydrophobic Ni−CoFe_2_O_4_ foam (**c**). LSV curves of the CoFe_2_O_4_ composites (**d**). The inset of Figure 4b shows the surface temperature of the pure water surface and the device given by the infrared camera. Inset of Figure 4d shows the phenomenon of oxygen production in the working electrode.

Different from the traditional heating effect of solar energy on the whole amount of water, interface heating only works on a small amount of water, improving the efficiency of steam production. A photo of the device is shown in Figure 4a. To verify the improvement in the steam production efficiency, a comparative experiment was performed. As shown in Figure 4b, the three-layer device had the highest steam production rate (1.68 kg·m^−2^·h^−1^) compared with pure water, the superhydrophobic Ni-CoFe_2_O_4_ foam and the superhydrophobic Ni-CoFe_2_O_4_ foam/filter paper composite. Experiments on water drops and friction stability were designed to verify the stability of the superhydrophobic Ni-CoFe_2_O_4_ foam. The water drop was 3 cm above the surface of the superhydrophobic Ni-CoFe_2_O_4_ foam. In the friction test, the sample size was 1.5 cm × 1.5 cm, and the load was 130 g. Sandpaper served as a friction surface. After the dripping of 1000 droplets and a friction test for 200 cycles, a contact angle of greater than 150° remained on the surface of the superhydrophobic Ni-CoFe_2_O_4_ foam, demonstrating an excellent superhydrophobic stability (Figure 4c). In addition, the CoFe_2_O_4_ foam composite prepared could be used as a water oxidation electrocatalyst. A three-electrode system (the sample was taken as the working electrode, a saturated calomel electrode was used as the reference electrode and a graphite rod was used as the counter electrode) and an electrolyte of 1 M KOH were used in the LSV test. The conversion between the saturated calomel electrode (*SCE*) and the *RHE* was calculated through the following formula:

(1) E(RHE)=E(SCE)+0.059pH+0.241

Due to the 1M KOH solution used in the testing process, the pH was 14. As shown in Figure 4d, the starting potential of the Ni-CoFe_2_O_4_ foam was less than that of the Ni foam, and the phenomenon of oxygen production was observed in the working electrode. Through device design, not only the removal of the floating organic solvent and clean water production, but also the multifunctionality of the device was realized.

## 4. Conclusions

In this paper, CoFe_2_O_4_ was grown on Ni foam through hydrothermal synthesis, and a superhydrophobic Ni-CoFe_2_O_4_ foam was prepared through silane modification. CoFe_2_O_4_ had a scaly morphology, and the crystal structure of CoFe_2_O_4_ was not changed after the hydrophobic modification. CoFe_2_O_4_ was proved to be the mixture valence state of Co^2+^, Co^3+^, Fe^2+^ and Fe^3+^. The contact angle of the superhydrophobic Ni-CoFe_2_O_4_ foam reached 157°, which had an excellent selective organic solvent removal property. The device consisting of a superhydrophobic Ni-CoFe_2_O_4_ floating layer, a hydrophilic layer and a photothermal layer could reach a steam generation rate of 1.68 kg·m^−2^·h^−1^. The hydrophobic stability of the superhydrophobic Ni-CoFe_2_O_4_ foam was demonstrated through water dropping and friction experiments.

## Data Availability

The data used to support the findings of this study are included within the article.

## Supplementary Materials

The following are available online at https://www.mdpi.com/article/10.3390/nano12091531/s1, Table S1: Relevant information of the XPS spectrum.
