# Supplementary material for: Design of Superhydrophobic CoFe2O4 Solar Seawater Desalination Device and Its Application in Organic Solvent Removal
Source: Nanomaterials (Basel). 2022 May 2;12(9):1531. doi: 10.3390/nano12091531 (PMC9104273; doi:10.3390/nano12091531)
Supplement: Supplementary file 1 [file nanomaterials-12-01531-s001.zip › nanomaterials-1679281-supplementary.pdf]

Communication

Design of Superhydrophobic $CoFe_2O_4$ Solar Seawater Desalination Device and Its Application in Organic Solvent Removal

Xiangcai Ge [1], Zhijun Zhou [1], Zheng Tan [1], Shoufei Wang [1], Xingchuan Zhao [1,*], Guina Ren [2], Bo Ge [1,*] and Wei Li [1]

[1] School of Materials Science and Engineering, Liaocheng University, Liaocheng 252059, China; gexiangcai@lcu.edu.cn (X.G.); 2020205938@stu.lcu.edu.cn (Z.Z.); 2019205643@stu.lcu.edu.cn (Z.T.); 2018206124@stu.lcu.edu.cn (S.W.); liwei@lcu.edu.cn (W.L.)
[2] School of Environmental and Material Engineering, Yantai University, Yantai 264405, China; guina.ren@ytu.edu.cn
* Correspondence: zhaoxingchuan@lcu.edu.cn (X.Z.); gebo@lcu.edu.cn (B.G.)

Citation: Ge, X.; Zhou, Z.; Tan, Z.; Wang, S.; Zhao, X.; Ren, G.; Ge, B.; Li, W. Design of Superhydrophobic $CoFe_2O_4$ Solar Seawater Desalination Device and Its Application in Organic Solvent Removal. *Nanomaterials* **2022**, *12*, 1531. https://doi.org/10.3390/nano12091531

Academic Editor: Ana C. Perdigón

Received: 29 March 2022
Accepted: 28 April 2022
Published: 2 May 2022

Publisher's Note: MDPI stays neutral with regard to jurisdictional claims in published maps and institutional affiliations.

Copyright: © 2022 by the authors. Submitted for possible open access publication under the terms and conditions of the Creative Commons Attribution (CC BY) license (https://creativecommons.org/licenses/by/4.0/).

Table S1 Relevant information of the XPS spectrum

Name	Binding energy
Co^{2+}	782 eV /795.6 eV
Co^{3+}	780.2 eV /787.9 eV
Fe^{2+}	718.9 eV
Fe^{3+}	710.7 eV/713 eV/725.1 eV
